# Endothelial Homeostasis Under the Influence of Alcohol—Relevance to Atherosclerotic Cardiovascular Disease

**DOI:** 10.3390/nu17050802

**Published:** 2025-02-26

**Authors:** Yusof Gusti, Weimin Liu, Fathima Athar, Paul A. Cahill, Eileen M. Redmond

**Affiliations:** Department of Surgery, University of Rochester Medical Center, Rochester, NY 14642-8410, USA; yusof_gusti@urmc.rochester.edu (Y.G.); weimin_liu@urmc.rochester.edu (W.L.); fathima_athar@urmc.rochester.edu (F.A.); paul.cahill@dcu.ie (P.A.C.)

**Keywords:** alcohol, ethanol, endothelium, vascular, atherosclerosis, cardiovascular disease, drinking, alcohol consumption, vessel health

## Abstract

Alcohol, in the form of ethyl alcohol or ethanol, is a widely consumed substance with significant implications for human health. Research studies indicate multifaceted effects of alcohol on the cardiovascular system with both protective and harmful effects on atherosclerotic cardiovascular disease (ASCVD), depending on the amount involved and the pattern of consumption. Among the critical components of the cardiovascular system are endothelial cells which line blood vessels. These cells are pivotal in maintaining vessel homeostasis, regulating blood flow, and preventing thrombosis. Their compromised function correlates with arterial disease progression and is predictive of cardiovascular events. Here we review research investigating how alcohol exposure affects the endothelium to gain insight into potential mechanisms mediating alcohol’s influence on ASCVD underlying heart attacks and strokes. Studies highlight opposite effects of low versus high levels of alcohol on many endothelial functions. In general, low-to-moderate levels of alcohol (~5–25 mM) maintain the endothelium in a non-activated state supporting vascular homeostasis, while higher alcohol levels (≥50 mM) lead to endothelial dysfunction and promotes atherosclerosis. These biphasic endothelial effects of alcohol might underlie the varying impacts of different alcohol consumption patterns on ASCVD.

## 1. Alcohol and Atherosclerotic Cardiovascular Disease

**Figure 1:**
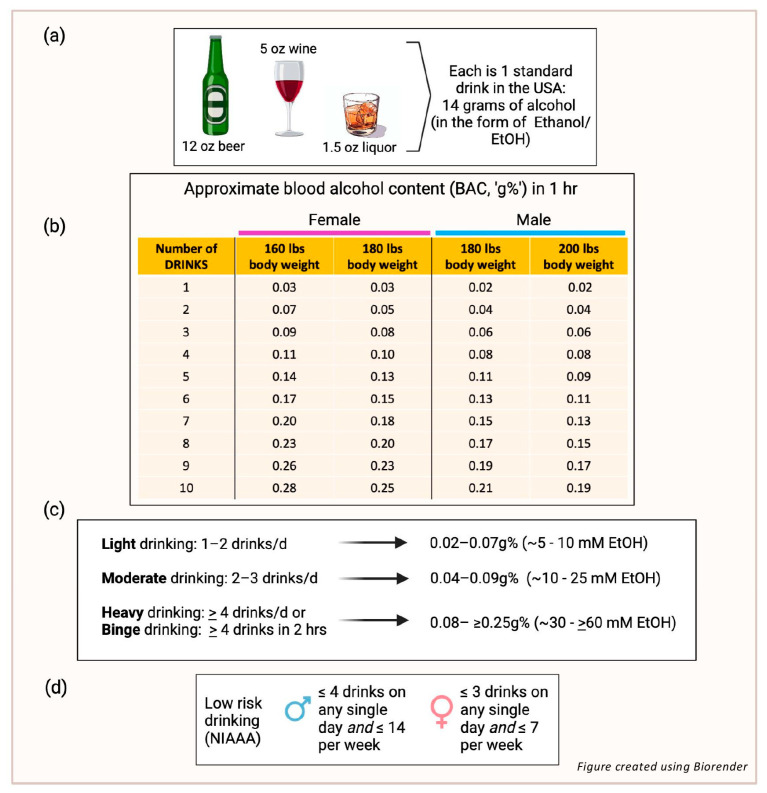
(**a**) A standard drink in the USA contains 14 g of ethyl alcohol (EtOH). (**b**) Blood alcohol concentration (BAC) is the measure of alcohol in the blood as a percentage; it is written as grams of alcohol per 100 ml of blood, or ‘g%’. The table shows BACs that might be achieved after consuming different numbers of drinks in men or women with the body weights indicated. A person with a BAC of 0.08 g% or higher is considered legally impaired to drive a vehicle. (**c**) Although definitions vary around the world, the number of drinks considered generally to represent light, moderate, and heavy/binge consumption are indicated together with expected BAC ranges and corresponding concentrations in millimolar (mM). (**d**) The National Institute on Alcohol Abuse and Alcoholism (NAAA) defines ‘low riskdrinking’ for men (up to 65 years old) as ≤4 drinks on any single day and ≤14 drinks/wk, andfor women as ≤3 on any single day and ≤7 drinks/wk.

Atherosclerotic cardiovascular disease (ASCVD) is a progressive disease of the arteries in which the vessel wall thickens and becomes less elastic as a result of pathologic remodeling and plaque build-up, ultimately restricting blood flow [1,2]. ASCVD underlies myocardial infarction/heart attack and ischemic stroke and is a major cause of mortality worldwide, accounting for almost a third of all deaths [3]. Risk factors for atherosclerosis include dyslipidemia, hypertension, and diabetes [2]. Of interest, alcohol (i.e., ethanol/(EtOH)) consumption in the form of beer, wine, or distilled spirits influences ASCVD [4,5,6]. Heavy drinking (i.e., four or more drinks per day) and/or bingeing (i.e., four or more drinks consumed in a short period of time) giving rise to blood alcohol concentrations (BACs) in the range 0.08 g–0.25 g% or ~30–≥60 mM EtOH (Figure 1) is harmful and increases cardiovascular disease risk. In contrast, numerous studies from the 1920s to the present—including observational studies, mouse experiments, small-scale human interventions, and in vitro human cell studies—suggest that light-to-moderate alcohol consumption (generally considered in the range of 1–3 drinks per day with BACs of 0.02 g–0.09 g% or ~5–25 mM EtOH) (Figure 1) may protect against ASCVD and enhance longevity [5,7,8,9,10,11,12]. While the latter association has been contested recently, including by those utilizing mendelian randomization approaches, the preponderance of evidence with respect to a protective effect on cardiovascular disease (ischemic heart disease and ischemic stroke) of light-to-moderate levels of alcohol, particularly in certain age groups, still holds [5,13,14]. Several factors can affect a person’s BAC (Figure 1), including the volume and concentration of alcohol consumed, the time taken to consume, whether taken with food or not, body weight and fat composition, metabolizing enzyme levels, sex and corresponding hormone levels, and certain medications [15].

## 2. Endothelium: Role in Atherogenesis

Endothelial cells line the intima of blood vessels and exhibit varied characteristics and responses to stimulation based on the type of vascular bed (e.g., artery vs. vein, large vessel vs. microcirculation) [16]. These cells play a critical role in arterial health and disease. As an interface between the blood and the vessel wall, the endothelium is ideally placed to sense changes in the circulatory milieu, and it acts as a gatekeeper of vessel health [17]. The endothelium is exquisitely responsive to changes in shear stress, i.e., the frictional force generated by flowing blood [18]. The normal functioning of the endothelium maintains vessel homeostasis, and its dysfunction and/or denudation is pivotal to the initiation, progression, and clinical complications of vascular disease [19,20]. In particular, a healthy endothelium has good barrier integrity, maintains optimum vascular tone, and ensures an anti-thrombotic, antioxidant, and anti-inflammatory interface. Conversely, an ‘activated’ or dysfunctional endothelium becomes leaky/more permeable, has reduced vasodilatory capacity and increased oxidative stress, and is pro-thrombotic and pro-inflammatory [20]. It is not surprising, then, that maintenance of endothelial function and/or the reversal of endothelial dysfunction are clinical goals and the foundation of some therapies under development for ASCVD.

## 3. Endothelial Barrier Function

A fundamental function of the endothelium is to act as a semipermeable barrier regulating the transport of macromolecules, bloodborne cells, and hormones between the vascular lumen and the vessel wall [19]. Many studies have examined the impact of alcohol on endothelial barrier function, primarily using EtOH concentrations of 50 mM to over 100 mM (i.e., 0.2 g% to over 0.4 g%) associated with heavy drinking, across various endothelial cell types. In general, exposure to high levels of EtOH compromised the integrity of the blood–brain barrier (BBB) and cerebral endothelium, known for their tight junctions, leading to junctional protein disorganization and triggering an inflammatory or oxidative response [21,22,23,24]. In keeping with these in vitro data, the BBB was found to be disrupted in postmortem alcoholic brains [25].

Fewer studies have investigated alcohol effects on arterial endothelium in the context of ASCVD and/or using lower doses of EtOH. Xu et al., using human umbilical vein endothelial cells (HUVEC) and bovine pulmonary artery endothelial cells (BPAEC) in vitro, reported barrier disruption with 50 and 100 mM EtOH, as assessed by decreased trans endothelial electrical resistance (TEER) [23]. A compromised barrier function of human coronary artery endothelial cells (HCAEC) was also reported at these high doses [26]. However, in contrast, exposure to levels of alcohol consistent with moderate consumption (i.e., 25 mM EtOH/0.1 g%) was found to enhance barrier integrity [26]. Moreover, co-treatment with this moderate EtOH dose prevented HCAEC barrier compromise induced by the pro-atherogenic protein serum amyloid A1 (SAA1), an effect that was Notch-signaling dependent [27]. These data indicate a biphasic dose-dependent effect of EtOH on endothelial barrier function with high levels disruptive, and moderate levels protective.

## 4. Vasoactive Substances

The endothelium regulates vascular tone and, thus, regional blood flow and maintains vessel homeostasis chiefly through the production and release of a number of vasoactive substances [17,28]. These substances influence the contraction/relaxation and quiescence/proliferation state of underlying vascular smooth muscle cells present in the medial compartment of blood vessels. They are critical for regulating blood flow, blood pressure, and tissue perfusion, and they are involved in processes such as inflammation, coagulation, and wound healing. Key vasoactive substances produced by endothelium include the vasodilators nitric oxide (NO) and prostacyclin (PGI_2_) and the vasoconstrictors endothelin-1 (ET-1) and angiotensin II (Ang II). An imbalance between these vasodilators and vasoconstrictors exemplifies endothelial dysfunction [28].

## 5. Nitric Oxide (NO)

The 1998 Nobel prize in Physiology and Medicine was awarded to Drs Furchgott, Ignarro and Murad for their discoveries in relation to nitric oxide (NO) as a signaling molecule in the cardiovascular system [29]. Nitric oxide is synthesized from L-arginine by the enzyme endothelial nitric oxide synthase (eNOS) and is pivotal in regulating vascular tone and blood pressure [30]. NO activates guanylyl cyclase to increase the second messenger molecule cyclic guanosine monophosphate (cGMP). NO not only relaxes SMCs and inhibits their proliferation, but it also prevents platelet adhesion and aggregation and leukocyte adhesion and migration into the arterial wall. Impaired NO production or availability is a significant hallmark of endothelial dysfunction [31]

Detailed analyses of alcohol effects on NO and its mechanism of action in isolated arteries and in cultured endothelial cells have been performed. Most studies in cells, rodents, and human subjects show a stimulatory effect of alcohol (EtOH 15–50 mM) on eNOS activity and NO production [26,32,33,34,35]. Kuhlmann et al., compared the effect of low-to-moderate (10–50 mM) and high levels of EtOH (100–150 mM) on NO synthesis in HUVEC and found low-to-moderate levels of EtOH stimulatory but high levels inhibitory [36]. These studies suggest either a dose-dependent stimulation of NO by alcohol or a biphasic effect, with low-to-moderate alcohol levels stimulating vasoprotective NO production and high levels of alcohol inhibiting it.

## 6. Prostacyclin and Thromboxane A2

Prostacyclin is a member of the prostanoid family, synthesized from arachidonic acid via the enzyme cyclooxygenase (COX) [37]. It is a potent vasodilator and inhibitor of platelet aggregation and prevents the progression of atherosclerosis. Prostacyclin promotes smooth muscle relaxation by increasing cyclic adenosine monophosphate (cAMP) levels, which, in turn, activates protein kinase A (PKA) and causes a reduction in intracellular calcium, leading to vasodilation [38]. Thromboxane (TXA_2_), also a product of the arachidonic acid cascade, is a powerful platelet activator and vasoconstrictor, and, thus, counteracts the actions of PGI_2_. An imbalance between the two, favoring TXA_2_, is indicative of endothelial dysfunction and is considered pro-atherogenic [39].

Consistent with its facial flushing/vasodilatory effects, the alcohol metabolite acetaldehyde is well known as a strong stimulator of PGI_2_ [40]. However, reports of the effect of alcohol on PGI_2_ are varied, with most studies indicating no effect, and a few suggesting a stimulatory effect at high doses (50–218 mM EtOH) [41,42,43]. However, studies also reveal that ethanol at moderate levels (22–30 mM EtOH) alters the PGI_2_/TXA_2_ balance in favor of PGI_2_, mediated exclusively by the inhibition of TXA_2_, an effect considered ultimately anti-atherogenic [40,44,45].

## 7. Endothelin-1

Endothelin-1 (ET-1), discovered in 1988 by Dr Yanagisawa’s group, is one of the most potent vasoconstrictors identified [46]. Many of the cardiovascular complications associated with aging and cardiovascular risk factors are initially attributable, at least in part, to an overproduction of ET-1 that results in an imbalance with its functional opposite, nitric oxide [47].

A search of the literature reveals that most studies investigating alcohol beverages and ET-1 focus on red wine components, rather than ethanol itself. These studies found reduced ET-1 production by endothelial cells in culture following exposure to red wine extracts, as well as decreased plasma ET-1 levels after red wine consumption (~4–6 glasses) [48,49,50]. We note that the effects of red wine, and of its associated polyphenols such as resveratrol, on endothelial function (including ET-1 effects) and on cardiovascular health has been previously reviewed in depth by others (e.g., [51,52,53]). As regards ethanol, Tirapelli et al., reported in a rat model that chronic (two weeks) heavy alcohol consumption, resulting in BAC ~180 mg/dL (i.e., ~45 mM), resulted in enhanced ET-1 induced-contraction in the rat carotid, a response that correlated with greater ETA receptor protein levels but reduced ETB receptor levels [54,55]. The effects of low levels of EtOH, however, were not investigated in that study.

Of note, in a human study, switching alcohol consumption from moderate-to-heavy (i.e, average 72 g EtOH/d to light (~8 g EtOH/d) over a four week period had no effect on plasma ET-1 levels [56]. Moreover, Di Gennaro et al. found that circulating ET-1 levels remained elevated (double that of controls), concomitant with impaired flow-mediated dilation (FMD) and increased blood pressure in a group of disease-free former alcoholics [57]. These data suggest that increased ET-1 levels and its associated endothelial dysfunction caused by chronic alcohol abuse persists even when alcohol consumption is reduced or stopped.

## 8. Angiotensin-II

The renin–angiotensin system (RAS) classically involved in blood pressure control variously affects cardiovascular disease pathogenesis [58,59]. The main effector peptide of the RAS is Angiotensin-II (Ang-II) that is formed following the cleavage of Angiotensin-I by angiotensin converting enzyme (ACE). Ang-II binds to either type 1 (AT1) or type 2 (AT2) receptors. Ang-II can be converted by ACE2 into angiotensin 1–7 (Ang-1–7) that mainly interacts with the Mas receptor (MasR). Importantly, the ACE/Ang-II/AT1 pathway drives atherosclerosis by promoting endothelial dysfunction and inflammation, and causing vasoconstriction partially by increasing the production of superoxide anions (O_2_^−^) via the activation of NAD(P)H oxidase [60]. Meanwhile, the ACE2/Ang-1–7/MasR and Ang1–7/AT2 pathways inhibit these processes and protect against atherosclerosis development [58].

Alcohol consumption has been linked to increased Ang II levels in the vasculature, leading to oxidative stress and endothelial dysfunction. Chronic heavy consumption (EtOH 4 g/kg daily for 12 weeks; BAC ~80 mM) caused hypertension in rats and elevated plasma and aortic Ang-II levels, concomitant with endothelial cell dysfunction with impaired vascular relaxation and depleted NO [61].

Similarly, Passaglia et al., and Awata et al., reported that following chronic alcohol intake (EtOH 20% *v*/*v* in drinking water, BAC ~40 mM, for 2–12 weeks following a 3-week ‘dose adaptation’ period), rodents had raised blood pressure, increased vascular oxidative stress, and decreased NO bioavailability, compared with controls, all effects that were prevented by Losartan, an AT1 receptor antagonist [62]. Ethanol treatment stimulated plasma Ang-I and Ang-II levels and plasma ACE activity [62]. This treatment also caused Ang-II-dependent arterial hypercontractility and induced mitochondrial dysfunction, effects blocked by Losartan [63]. These data from rodent studies indicate that the RAS and AT1 activation might contribute to the cardiovascular risks and endothelial dysfunction linked to heavy alcohol consumption. This suggests that AT1 receptor blockers may have a therapeutic role in protecting against vascular toxicity caused by alcohol abuse. The acute and chronic effects of low-to-moderate-dose alcohol (5–25 mM EtOH) on Ang II activity within arterial vasculature and the endothelium remain undetermined.

## 9. ROS/Oxidative Stress

Reactive oxygen species (ROS) are molecules with an unpaired electron such as superoxide anions (O_2_^−^), hydroxyl radical (•OH), or peroxynitrite (ONOO^−^). When maintained at low or moderate levels, ROS can be beneficial to health by facilitating cell signaling and maintaining vascular homeostasis [64,65]. However, an imbalance between the production and accumulation of ROS/free radicals and the ability of antioxidants (e.g., superoxide dismutase, glutathione peroxidase, catalase, vitamin E) to scavenge them results in oxidative stress that leads to cell damage [64,65]. Oxidative stress is causative of the onset and progression of atherosclerotic cardiovascular disease by driving inflammatory gene expression and stress signaling, triggering the release of cytokines and chemokines, inducing endothelial dysfunction, and reducing NO production [66,67].

Rajendran et al. reported a dose-dependent effect of alcohol on arterial endothelial ROS production, with both moderate- (25 mM) and high-dose (50 mM) EtOH exposures eliciting ROS generation but with a more pronounced response seen at the higher dose [26]. The exposure of these cells to acetaldehyde (10–25 μM) also stimulated ROS generation [26]. It was noted that the ROS stimulatory effects of ethanol were coincident with increased eNOS activity in these cells. Given that NO produced by eNOS acts as an antioxidant to counteract ROS [31], these two alcohol-induced responses might be expected to functionally cancel each other out regarding atherogenesis. Similarly, Haorah et al., demonstrated that EtOH (50 mM) exposure resulted in oxidative stress in BMVEC, leading to loss of barrier integrity and increased leukocyte migration [21].

As noted in recent reviews, studies in human subjects find increased ROS in binge drinking adults and those heavily consuming alcohol, suggesting that oxidative stress may be an important pathophysiological mechanism underlying vascular dysfunction in heavy drinkers (e.g., 5–6 drinks/d) [68,69]. However, several clinical studies suggest that low-to-moderate alcohol consumption (2–2.5 drinks/d), particularly when taken as red wine or beer, is associated with either no effect or a decrease in markers of oxidative stress [70,71]. Combining clinical and basic science studies, these data suggest that the type of alcoholic beverage, along with the duration and amount of consumption, significantly influences the relationship between alcohol intake and oxidative stress, where lower EtOH levels have minimal or inhibitory effects, while higher levels stimulate ROS generation.

## 10. Monocyte Recruitment and Adhesion

The chemokine monocyte chemoattractant protein-1 (MCP-1) binding to its receptor CCR2 facilitates monocyte recruitment and adhesion to the endothelium that is an important early step in atherosclerosis development [72]. Numerous studies involving animals, humans, and cell cultures have explored the impact of alcohol on MCP-1 and monocyte adhesion in relation to arterial disease, revealing a strong consensus for a biphasic response to alcohol. Badia et al. reported significantly less adhesion to TNFα-stimulated endothelial cells of monocytes isolated from moderate alcohol drinkers (two drinks/d, either red wine or gin over 28 days), compared to alcohol abstainers [73]. In a rabbit atherosclerosis model, moderate alcohol consumption significantly reduced MCP-1 levels in arterial segments [74]. Cullen et al. demonstrated that EtOH, over a wide range of doses (1–100 mM), inhibited interleukin-1β-stimulated MCP-1 production in HUVEC by decreasing gene transcription and mRNA stability [75]. Ethanol (~20–150 mM) inhibited tumor necrosis factor (TNF)-induced MCP-1 production and the adhesion of both monocytes and neutrophils to HMVECs [76]. MCP-1 expression and monocyte adhesion was stimulated following the exposure of HCAEC to the pro-inflammatory acute phase protein SAA-1, a known pathogen for atherogenesis [27]. These SAA-1-induced responses were strongly inhibited by co-treatment with moderate-level EtOH (25 mM), an effect mediated by Notch 1 signaling [27]. Moreover, a biphasic effect of alcohol itself on basal MCP-1 production and monocyte adhesion in HCAEC was noted, with moderate levels inhibitory and higher levels (≥50 mM) stimulatory [26]. Notably, exposure to the ethanol metabolite acetaldehyde (10–25 μM) increased CCR2 receptor expression in monocytes and, like high-level EtOH, enhanced monocyte adhesion to endothelial cells, an effect dependent on P-selectin and TNFα [77]. It is conceivable that after heavy binge drinking or chronic alcohol abuse, high levels of both ethanol and acetaldehyde may be simultaneously present and these could act synergistically to increase monocyte adhesion and worsen vascular inflammation.

## 11. Cell Adhesion Molecules

The increased endothelial surface expression of cell adhesion molecules (CAMs) such as vascular cell adhesion molecule 1 (VCAM-1) and intercellular adhesion molecule 1 (ICAM-1) (members of the immunoglobulin superfamily), and E-selectin (a lectin-like carbohydrate binding molecule), facilitates the transit of inflammatory monocytes to the sub-endothelium and is indicative of endothelial dysfunction [78]. CAM expression on endothelial cells is induced by cytokines and other stimuli [79]. VCAM-1 and ICAM-1 in particular play a crucial role in the development of atherosclerosis and can be found in early lesions [78]. Because of this, the effect of alcohol on these molecules is of interest and has been previously reviewed [80]. Studies reveal that treatment with high-dose alcohol (i.e., ≥50 mM EtOH) stimulates the endothelial expression of both VCAM-1 and ICAM-1 [26,76]. Plasma levels of VCAM-1, ICAM-1, and E-selectin were increased in rats following six weeks of heavy alcohol consumption (~50 mM EtOH/d) [81] and in chronic alcoholics vs. teetotalers [82]. In contrast, low-to-moderate-level alcohol treatment (≤25 mM EtOH) reduced endothelial VCAM-1 and ICAM-1 expression compared to controls, and in addition, prevented their inflammatory protein-induced expression via a Notch-signaling-dependent mechanism [26,27]. In sum, these data indicate differential effects of low vs. high dose alcohol on endothelial CAM expression, with low levels inhibitory and high levels stimulatory.

## 12. Inflammatory Cytokines and CRP

Suppression of inflammation is a promising therapy for atherosclerosis [83]. This is due, in no small part, to the fact that inflammatory cytokines are key instigators of endothelial dysfunction and arterial pathology [84,85]. Specifically, cytokines such as tumor necrosis factor-α (TNF-α), interleukin-1beta (IL-1β), interferon-gamma (IFNγ), interleukin-6 (IL-6), and transforming growth factor-beta (TGFβ) disrupt vascular homeostasis in a multipronged way, including by (i) promoting oxidative stress and reducing NO bioavailability, (ii) recruiting monocytes to the site of endothelial injury, (iii) activating T cells and macrophages to amplify the local inflammatory response, and (iv) stimulating medial smooth muscle cells to proliferate and migrate, contributing to neointimal development [85]. In addition to endothelial cells, vascular smooth muscle cells and macrophages can also produce inflammatory cytokines in response to various stressors, which can then act on the cell of origin in a feedback loop [85]. For example, both endothelial cells and smooth muscle cells respond to stimulation with IL-1 or TNFα by producing large amounts of IL-6, itself a major stimulator of C-reactive protein (CRP) a classical acute phase protein and a systemic marker of inflammation [86,87].

Researchers have investigated the effect of alcohol on inflammatory cytokines in the context of atherosclerosis. While heavy alcohol use is believed to be pro-inflammatory [88], studies suggest that low-to-moderate levels may have the opposite effect, being anti-inflammatory. Indeed, anti-inflammatory effects have been considered as a possible explanation of the way in which moderate alcohol could lower the risk of ASCVD. In active older adults, light-moderate alcohol consumption (1–7 drinks/wk) was associated with a reduced risk of all-cause mortality and a 30% reduced risk of cardiac events, concomitant with lower levels of IL-6 and CRP, but not TNFα [89]. Of note, baseline levels of CRP are strong independent predictors of future risk of heart attack and stroke [90]. Lower levels of CRP have been consistently found in moderate alcohol consumers (1–2 drinks/d) compared to abstainers and heavy drinkers (≥5 drinks/d) [91].

Marques-Vidal et al. investigated the association between alcohol consumption and inflammatory chemokines in a population of ~6000 men and women. They found that moderate alcohol consumption (1–2 drinks/d) was associated with lower levels of IL-6 and TNFα (with no effect on IL-1β) [92]. Lassaletta et al., demonstrated, in a swine model of chronic myocardial ischemia, that moderate alcohol consumption (~2 drinks/d) significantly improved arteriolar density and myocardial perfusion, concomitant with significantly decreased cytokine IL-8 levels [93]. Moreover, there was a j-shaped relationship reported between alcohol exposure and IL-6 and IFNγ production in human arterial endothelial cells, with low-moderate levels (10–25 mM EtOH) inhibiting and higher levels (≥50 mM EtOH) stimulating [26]. Thus, there is considerable evidence from different types of studies to support biphasic inflammatory-modulating effects of alcohol expected to impact ASCVD development.

## 13. Endothelial Control of Thrombosis and Fibrinolysis

Under physiological conditions, the endothelium produces several factors that inhibit platelet activation and adhesion. In addition to the vasoactive substances PGI_2_ and NO, as mentioned previously, these factors include anti-thrombotic proteins like thrombomodulin, CD39 (an ectonucleotidase), and plasminogen activators [19].

Thrombomodulin is a glycoprotein receptor for thrombin on endothelial cells. The thrombin–thrombomodulin complex activates protein kinase C, which proteolyzes several coagulation factors, thereby inhibiting coagulation [94]. The proinflammatory cytokine TNFα enhances the pro-coagulant properties of endothelial cells primarily by decreasing thrombomodulin activity [95]. Given that moderate alcohol consumption reduces TNFα levels and inflammation [92], alcohol may indirectly increase the anti-thrombotic thrombomodulin activity of endothelial cells in this way [96,97].

CD39 is the main endothelial ectonucleotidase and a key modulator of thrombus formation and is, thus, important to sustaining vascular homeostasis [98,99]. It is also expressed on platelets and smooth muscle cells. CD39 hydrolyzes ATP and ADP to generate adenosine that is both anti-inflammatory and anti-thrombotic [98]. Platelet CD39 activity was reportedly increased following red wine and grape juice consumption in diabetic rats [100,101]. CD39 levels in the livers (in vivo) and Kuppfer cells (in vitro) of ethanol-fed mice were increased compared to controls [102]. However, little to no information exists in the literature with respect to effects of alcohol on endothelial CD39 expression or activity in the context of ASCVD.

Plasminogen activators (PAs) decrease fibrin-based thrombosis by increasing fibrinolysis [103]. Surface-localized endothelial fibrinolytic activity contributes to vascular homeostasis and involves tissue-type plasminogen activator (t-PA) as well as urokinase-type plasminogen activator (u-PA) and its receptor uPAR [103,104]. Plasminogen activator inhibitor-1 (PAI-1) is the main regulator of uPA and tPA [104]. Annexin II, abundantly expressed on endothelial cells, is a receptor for plasminogen activator and plasminogen [105]. Researchers have determined alcohol effects on the plasminogen fibrinolysis system. Brief exposure (~30 min) of cultured endothelial cells to low levels of alcohol (0.01–0.1 g%/2.5–25 mM EtOH) increased plasminogen receptor activity, the expression of annexin II and plasminogen activator mRNA, and decreased PAI-1 mRNA levels, thus overall increasing fibrinolytic activity [106,107,108,109]. In contrast, following consumption by male volunteers of a large amount of alcohol (several glasses of red wine), there was a marked increase in PAI-1 and an inhibition of fibrinolysis as determined by clot lysis assay [110,111]. Together, these data point to a potential biphasic effect of alcohol on plasminogen-mediated fibrinolysis, with low doses pro-fibrinolytic and high doses anti-fibrinolytic.

## 14. Endothelial Repair/Endothelial Progenitor Cells

In large part, the balance between the dysfunction and regeneration of the arterial endothelium dictates the risk for ASCVD. Endothelial progenitor cells (EPCs) are bone-marrow-derived cells circulating in peripheral blood, defined as expressing both a stem cell marker (e.g., CD34, CD133) and an endothelial protein (e.g., KDR/VEGFR2) [112,113]. The roles of EPC in endothelial repair in the context of arterial disease and de novo blood vessel formation remain incompletely understood. Once thought to mobilize, differentiate and incorporate at the site of endothelial injury after homing there, it is now appreciated that EPC-derived paracrine signals might also orchestrate the repair process [114]. In the latter scenario, when the endothelium is injured, circulating EPCs are induced to secrete various cytokines and pro-angiogenic growth factors such as VEGF, stromal cell derived factor 1 (SDF-1), and NO, resulting in the production of an angiogenic microenvironment that stimulates the nearby endothelium to proliferate [115]. Moreover, EPC-derived extracellular vesicles have abundant biological activity and are believed to be a stable source of exosomes promoting vascular endothelial repair [116,117]. Circulating levels of EPCs are thought to correlate with atherosclerotic burden and with in-stent restenosis after percutaneous coronary intervention, and thus, they are broadly considered as biomarkers that reflect the integrity and repair capacity of the endothelium and cumulative cardiovascular disease [118]. The number of circulating EPCs and their functional capacity (i.e., their replicatory and migratory properties) has been reported in some, but not all, studies to correlate inversely with risk factors for ASCVD such as age, smoking, hypertension, diabetes mellitus, and hyperlipidemia [115].

Researchers have investigated the possible role of circulating EPC in mediating the effects of alcohol on ASCVD—particularly, the beneficial effect of moderate alcohol, with mixed results. Neither alcohol consumption, nor smoking or physical activity, had any effect on the number of circulating EPCs in healthy adults (men and women, mean age 35) [119]. While beer and non-alcoholic beer increased the number of circulating EPCs and stromal cell derived factor 1 (SDF1) levels in a cohort of ‘high cardiovascular risk’ men, ethanol itself (in the form of gin, a distilled beverage lacking polyphenols) (30 g/d) had no effect [120]. However, in the population-based Bruneck study, the EPC number was positively correlated with alcohol consumption (13–22 g EtOH/d) [121], but the type (wine vs. distilled spirits vs. beer) was not delineated. In mice, the intake of red wine (100 mL/d for three weeks) increased the number and functional capacity of circulating EPCs by enhancing NO bioavailability, but these effects were not seen with beer (250 mL/d) or with vodka (30 mL/d) [122].

In an in vitro study, alcohol at low-to-moderate concentrations (17–34 mM EtOH) increased the angiogenic functional capacity of EPCs as determined by measuring the migration, tube formation, and proliferation of endothelial colony forming cells, a proliferative subtype of EPCs. This effect was associated with the increased expression of VE-cadherin that is important for cell–cell interaction and endothelial repair capacity [123].

In a mouse model of atherosclerosis (i.e., Ang II-infusion in APOe knockouts) moderate-dose EtOH (10 mg/d, IP) inhibited, whereas high-dose EtOH (30 mg/d, IP) exacerbated, atherosclerotic lesions compared to controls [124]. The stimulation of SDF-1 levels and maintenance of circulating EPCs at relatively high levels were thought to explain the anti-atherosclerotic effect of moderate-dose ethanol in that study [124]. In a high-fat diet ‘metabolic disease’ mouse model, low-dose EtOH (20 mM) activated bone marrow-derived EPCs as determined by their increased angiogenic activity and enhanced secretion of angiogenic cytokines compared to controls or to those treated with higher-dose EtOH (34 mM) [125]. Collectively, these data point to the possibility that while low-to-moderate-level alcohol might not affect the generation of circulating EPCs, it might play a role in their functional activation.

## 15. Endothelial Notch Signaling

Multiple pathways traditionally associated with embryonic development, such as BMP-TGFβ, WNT, and Notch, are now recognized as shear stress-regulated factors that play a key role in atherosclerosis in the adult [83,126]. For example, endothelial Notch signaling is mechanosensitive and may impact atherogenesis by modulating immunity [127] and by influencing endothelial cell quiescence, junctional stability, and phenotype [128]. The canonical signaling interaction of the Notch receptor (Notch1–4) with a ligand (Delta-like 1, -3, or -4, or Jagged 1 or 2) initiates the sequential proteolytic cleavage of the receptor by α-secretase followed by γ-secretase, resulting in the release of the Notch intracellular domain (NICD) from the cytoplasmic side of the cell membrane [129]. The NICD is translocated into the nucleus, where it binds to transcription factor RBP-J and forms a ternary complex with co-activators including Mastermind-like-1 (MAML-1), resulting in the transcriptional activation of Notch target genes such as Hes and Hrt [129]. Notch signaling can also function through a less-well-characterized ‘non-canonical’ pathway that is independent of canonical ligand binding and γ-secretase cleavage [130].

A search of the literature did not find any studies detailing alcohol effects on BMP or WNT in the context of endothelial function/atherosclerosis. However, investigators have identified canonical Notch signaling as a novel target for alcohol in the vasculature. Alcohol differentially affects γ-secretase activity and, thus, Notch signaling in vascular smooth muscle and endothelial cells [131,132]. Specifically, in contrast to its inhibitory effect in vascular smooth muscle, alcohol at moderate levels (25 mM EtOH) stimulates γ-secretase activity and promotes Notch signaling in HCAEC [132,133]. Moreover, alcohol at higher doses (≥50 mM EtOH) did not stimulate Notch in these cells, suggestive of a hormetic effect (i.e., different effects of low vs. high doses) of alcohol on Notch signaling in the endothelium [26]. These differential alcohol-modulating effects on Notch signaling in vascular smooth muscle and endothelial cells might act in concert to mediate the varied effects seen on ASCVD in drinkers with different consumption behaviors.

## 16. Conclusions

**Figure 2:**
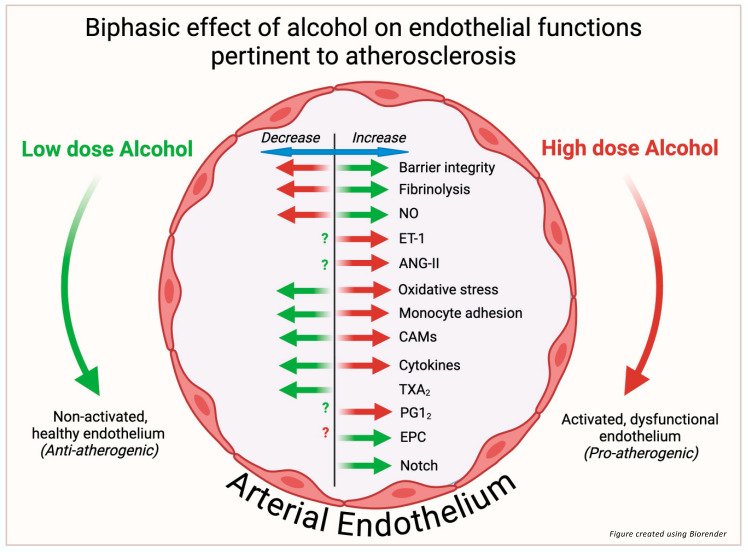
Low dose alcohol (5–25 mM EtOH) (green arrows) increases endothelial barrier integrity, fibrinolytic activity, nitric oxide (NO) production, Notch signaling and endothelial progenitor cell (EPC) functional activity, while it decreases oxidative stress, monocyte adhesion, inflammatory cytokine production, cell adhesion molecule (CAM) expression, and thromboxane (TXA_2_) levels. In contrast, high dose alcohol (≥50 mM EtOH) (red arrows) decreases barrier integrity, fibrinolysis and NO production, while increasing endothelin-1 (ET-1), angiotensin II (Ang-II), oxidative stress, monocyte adhesion, CAMs, inflammatory cytokines and prostacyclin (PGI_2_). Thus, low dose alcohol maintains the endothelium in a non-activated state that is anti-atherogenic, whereas high dose alcohol causes endothelial dysfunction that is pro-atherogenic. Such biphasic endothelial responses elicited by alcohol might underlie the differential effects of alcohol consumption on atherosclerotic cardiovascular disease (ASCVD) reported epidemiologically. [‘?’ indicates not determined].

There is abundant experimental evidence demonstrating that alcohol exposure affects many important functions of endothelial cells that would be expected to impact the initiation and development of atherosclerotic cardiovascular disease. These include effects on barrier integrity, vasoactive substances, oxidative stress, inflammation, fibrinolysis, monocyte recruitment and adhesion, endothelial progenitor cell functionality, and Notch signaling (Figure 2). From a review of the literature, the majority of studies focus on relatively high or very high alcohol doses (50–400 mM range). In far fewer instances, the effects of low-to-moderate doses (5–25 mM) of alcohol were investigated. When a range of doses was tested, a biphasic effect of alcohol was often found such that low-to-moderate levels (consistent with light-to-moderate drinking) maintained endothelial homeostasis, whereas higher levels (consistent with heavy consumption and bingeing) caused endothelial dysfunction. Thus, alcohol displays hormesis with respect to the vascular endothelium, with lower-dose effects predicted to be anti-atherogenic and higher-dose effects predicted to be pro-atherogenic. The biphasic effect of alcohol on endothelial cells highlights the importance of investigating alcohol doses modeling both light and heavy drinking, as well as underscoring the value of carefully detailing the doses and exposures used to facilitate comparison between studies. While some signaling pathways targeted by alcohol have been identified (e.g., Notch), detailed mechanistic information as to precisely how alcohol elicits its effects, particularly its low-dose effects, is still lacking and warrants further exploration. The investigation of the effect of alcohol on endothelial cell plasticity, endothelial extracellular vesicles, and microRNAs (all of which have been implicated in atherogenesis) might also yield novel mechanistic information. In any case, the biphasic endothelial responses elicited by alcohol and detailed herein likely account, at least in part, for the effects of various drinking patterns on ASCVD reported epidemiologically.
